# Reversal of Acute Lead Encephalopathy in a Child

**DOI:** 10.7759/cureus.15155

**Published:** 2021-05-21

**Authors:** Swasti Keshri, Anil Kumar Goel, Ankit Kumar Garg

**Affiliations:** 1 Paediatrics Emergency Medicine, All India Institute of Medical Sciences, Raipur, IND; 2 Paediatrics, All India Institute of Medical Sciences, Raipur, IND; 3 Orthopaedics, All India Institute of Medical Sciences, Raipur, IND

**Keywords:** cana2edta, d-penicillamine, lead encephalopathy, lead poisoning, lead toxicity

## Abstract

Lead poisoning, fairly common in the 20th century, has decreased drastically in the last decade. Severe lead poisoning in the form of encephalopathy has a fatality rate of 28% to 45% and neurological sequelae in about 82%. We present the management of a case of lead encephalopathy that recovered without any significant neurological sequelae in a resource-limited setting. A previously healthy seven-year-old boy presented with complaints of falling unconscious on the ground while playing, followed by multiple episodes of seizures, vomiting, and altered sensorium. The patient had pallor, Glasgow coma score of E2V3M3, with features of raised intracranial pressure. Lead poisoning was suspected as the patient had four months of exposure to a battery recycling factory. Management of seizures and raised intracranial pressure was done. X-ray long bones showed lead lines at the metaphysis. Blood lead levels were highly elevated (139.96 mcg/dL). Investigations revealed iron deficiency anemia, vitamin D deficiency, and renal tubular injury in the form of proteinuria. D-penicillamine with supplements was started due to unavailability of other chelating agents. Encephalopathy improved, but patient had psychiatric symptoms of hallucinations and delusions. On the 12th day, CaNa_2 _EDTA was started, which resulted in significant improvement in the psychiatric symptoms. The patient had near-complete recovery in another one month, the patient being able to read, write, recite and speak as the pre-illness state. In conclusion, lead poisoning remains a significant health problem even today. Early recognition and management are of paramount importance in its outcome.

## Introduction

Lead is a heavy metal that is toxic to the body even in low concentrations [1]. The major sources of lead are petroleum and gasoline products, lead-based paints, food cans, lead pipes, batteries, lead-containing herbal and traditional medicines, etc [1,2]. Plenty of cases of lead poisoning were reported in the industrial era from 1965 to 1990. However, recognition of lead as a health hazard and adoption of several lead poisoning prevention policies in the 90’s such as elimination of lead from gasoline products have drastically decreased the cases of lead poisoning in the 21st century, with very few cases being reported from “lead hot-spots” like battery recycling plants, refineries, mines, etc. in the last decade [3]. Lead can cause irreversible damage to children’s developing brain causing neurobehavioral changes, lower IQ, autistic behaviour, conduct disorder, delinquency, criminal behaviour, seizures, and encephalopathy in severe cases [3]. It also causes immune dysfunction, anaemia, gastrointestinal symptoms, etc., at a much lower level than in adults, making children biologically more susceptible than adults [2]. Lead in higher levels can present as encephalopathy, which has a mortality rate of 28% to 45%, with as many as 82% having neurological sequelae [4], hence it is critical that paediatricians and general practitioners are aware of the clinical presentation and management of lead poisoning. This case highlights that although not reported, lead poisoning remains a significant issue even today, particularly in the industrial belts of the country, and its early recognition is of paramount importance.

## Case presentation

A seven-year-old boy, third in birth order of a non-consanguineous marriage, with normal development (student of first standard), normal nutrition, complete immunisation, insignificant past history, presented with complaints of falling unconscious on the ground while playing in a circular motion, followed by tonic posturing of all four limbs with up rolling of eyeballs lasting for about five minutes, followed by multiple episodes of vomiting and altered sensorium. The patient had no history of fever, jaundice, ear discharge, bleeding diathesis, chest pain, palpitations, breathing difficulty, decreased urine output, diarrheal illness, drug intake, or animal bite. On examination, patient was afebrile, heart rate of 92/min, blood pressure between 50th and 90th centile, respiratory rate of 26/min, blood sugar of 92 mg/dl, had some pallor, and was in an altered sensorium (Glasgow coma score of E2V3M3), with features of raised intracranial pressure in the form of increased tone and brisk deep tendon reflexes and presented with multiple episodes of generalised tonic-clonic seizures in the emergency. The rest of the systemic findings were normal. Differentials of post-traumatic intracranial haemorrhage, cerebral contusion, post-traumatic diffuse cerebral oedema, status epilepticus, and intracranial space-occupying lesions were kept.

**Figure 1:**
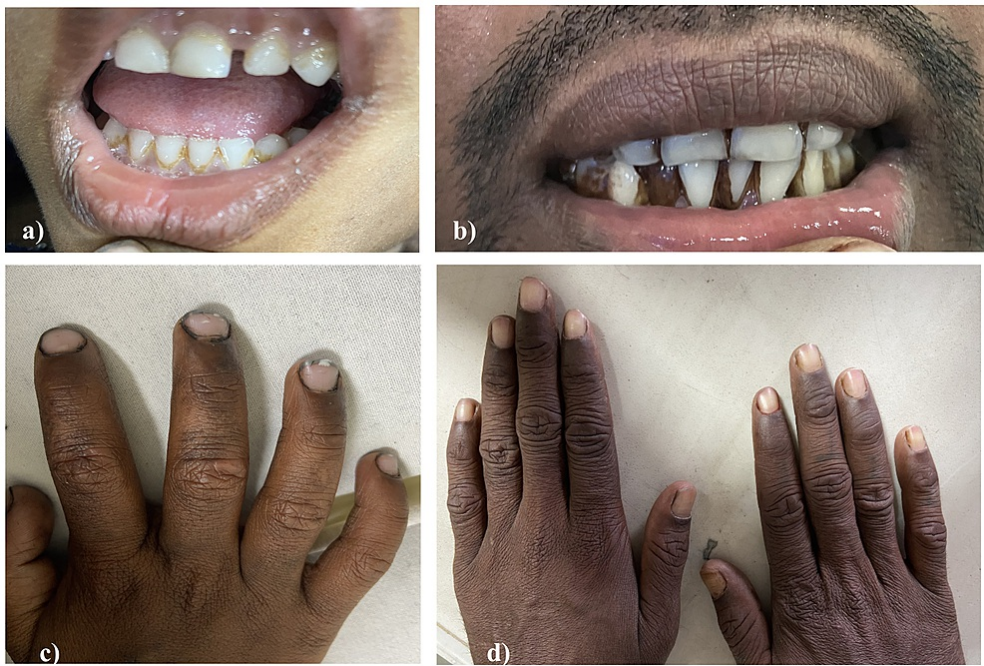
Clinical pictures (a, c: patient; b, d: father). The patient’s father had bluish discolouration at the junction of gums and teeth (Bruton’s line) and white lines of discolouration across the fingernails (Mees lines), which was not present in the patient himself.

Management of seizures as per status-epilepticus protocol (phenytoin, valproate, levetiracetam, midazolam infusion), intracranial pressure with 3% hypertonic saline, and supportive treatment was done. The non-contrast CT brain was normal with normal differentiation of the grey-white matter. The initial blood investigations of complete blood count, electrolytes sodium, calcium, magnesium, renal function tests, liver function tests, coagulation profile were unremarkable except for microcytic, hypochromic anaemia. As an etiological diagnosis could not be established, a repeat history was taken on day 2, which revealed that the child had eight hours a day of four-month exposure to a car battery recycling factory, where his parents were employed. Lead poisoning was strongly suspected, and the history of its symptoms was re-evaluated. The patient had a history of poor hand hygiene and poor overall hygiene. However, there was no history of pica, behavioural abnormalities, poor performance in school, constipation, anorexia, abdominal pain, fatigue, weight loss, or paresthesia of fingers and toes. The patient’s father had bluish discolouration at the junction of gums and teeth (Bruton’s line) and white lines of discolouration across the fingernails (Mees lines), which was not present in the patient himself (Figure 1).

**Figure 2:**
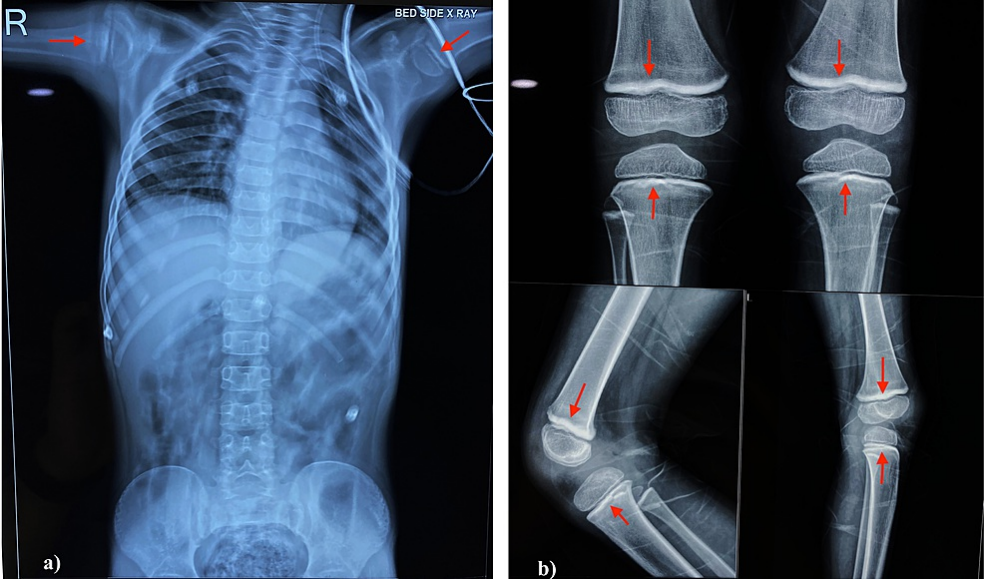
X-ray abdomen and long bones. (a) X-ray abdomen did not reveal any radio-opaque lead flecks. (b) X-ray of long bones (femur, tibia, humerus) showed lead lines at the metaphyseal ends.

X-ray of long bones (femur, tibia, humerus) showed lead lines at the metaphyseal ends (Figure 2).

X-ray abdomen did not reveal any radio-opaque lead flecks (Figure 2). Blood lead levels (BLLs) were highly elevated (139.96 mcg/dl), thereby establishing a diagnosis of lead poisoning (Table 1).

**Table 1:** Investigations of the patient.

Parameters	Patient’s value	Reference range
Blood lead levels (BLL)	139.96 mcg/dL	<5 mcg/dL
Hemoglobin	9.2 g/dL	>11 g/dL
Hematocrit	28.7%	35-45%
RBC	3.79 x 10^6^/mL	4.0-5.2 x 10^6^/mL
Mean corpuscular volume (MCV)	75.7 fL	77-95 fL
Mean corpuscular hemoglobin (MCH)	24.3 pg	25-33 pg
Mean corpuscular hemoglobin concentration (MCHC)	32.1 g/dL	31-37 g/dL
Red cell distribution width (RDW)	16.7%	11.6-14.0%
Corrected reticulocyte count	1.3%	0.5-1.5%
Total leucocyte count	11.2 x 10^3^/mL	5-13 x 10^3^/mL
Platelet count	335 x 10^3^/mL	150-450 x10^3^/mL
Serum iron	11.67 mcg/dL	33-193 mcg/dL
Serum ferritin	7.5 ng/mL	7-140 ng/ml
Total iron-binding capacity (TIBC)	280 mcg/dL	250- mcg/dL
C- reactive protein	7.77 mg/L	<10 mg/L
Urine glucose	Not detected	Not detected
Spot urine protein	8.38 mg/dL	
Spot urine creatinine	17.2 mg/dL	
Spot urine protein/ creatinine ratio	0.487	<0.2
Urine routine- microscopy	No pus cells/ RBCs	No pus cells/RBCs
Serum creatinine	0.5 mg/dL	<0.7 mg/dL
Serum vitamin D levels	10.82 ng/mL	30-100 ng/mL
Serum calcium	9.0 mg/dL	9.3-10.6 mg/dL
Serum magnesium	1.9 mg/dL	1.7-2.7 mg/dL
Serum phosphorus	3.4 mg/dL	4.4-6.0 mg/dL

Peripheral blood smear showed microcytic, hypochromic anaemia without basophilic stripling or features of haemolysis. Iron studies revealed iron deficiency anaemia. The patient also had vitamin D deficiency, hypocalcemia, and hypophosphatemia. Patient had normal urine output and serum creatinine level, but the spot protein/ creatinine ratio was 0.48, suggestive of non-nephrotic proteinuria and mild renal tubular dysfunction. With symptomatic management of seizures, raised intracranial pressures, and supportive management, encephalopathy improved (E4V4M6), and seizure episodes stopped by the third day. Despite establishing a diagnosis of acute lead toxicity by day three, due to the unavailability of dimercaprol/ CaNa2EDTA/ succimer, chelation therapy could not be started. Third-line chelating agent, D-penicillamine (20 mg/kg/day), zinc, vitamin c, calcium, and vitamin D supplements were started. By day 6, encephalopathy improved, but the patient had psychosis in the form of delusion of persecution, auditory and visual hallucinations. The patient had aggressive behaviour, biting, shouting, inappropriate smiling, pointing out objects, seeing snakes and insects crawling over him, hearing voices not heard by others, and being fearful of those voices. Risperidone and clonazepam were added as per psychiatric consultation. The psychosis improved gradually. Due to resource-limited settings, dimercaprol could not be procured, and CaNa2 EDTA was given at 1,500 mg/m^2^/day for three days on the 12th day. There were no side effects of the chelating agents. By the 16th day, following the administration of CaNa2EDTA, there was a tremendous improvement. Hallucinations, delusions, and aggressive behaviour resolved, and the patient only had cognitive impairment and inappropriate smiles. Repeat BLL and BLL of parents could not be done due to the unwillingness and financial constraints of the parents. The patient had near-complete recovery in another month, with resolution of all psychiatric symptoms, the patient being able to read, write, recite and speak as the pre-illness state. The case was also reported to the concerned authorities.

## Discussion

Centers for Disease Control and Prevention (CDC), 2012 defines no “safe” or “non-toxic” blood lead levels (BLLs) with the 97.5th percentile of BLL being at 5 mcg/dL and severe toxicity above 70 mcg/dl [1]. In children, lead is mostly absorbed through the gastrointestinal tract by ingestion, and less commonly by inhalation of burning lead-containing materials like batteries [2]. In our case, both routes might have attributed to the poisoning. Lead has the ability to substitute cations like calcium and zinc, thus altering signaling molecules in the membrane ionic channels, and can inhibit the function of N-methyl-D-aspartate type glutamate receptors, thus causing neurotoxicity as present in our case [2]. Our patient presented with anemia as lead causes inhibition of enzymes delta-aminolevulinic acid dehydratase and ferrochelatase causing inhibition of heme synthesis [2]. The iron deficiency and vitamin D deficiency must have increased the gastrointestinal absorption of lead, causing increased toxicity [2]. Basophilic stripling, caused by aggregation of altered ribosomes due to high BLL, was absent in our case, and may be missed commonly. Our patient also presented with renal tubular damage in the form of proteinuria, which may be a presenting symptom [2]. However, in our case, “lead colic” - constipation, abdominal pain, and vomiting, which is a common feature of severe lead poisoning, was absent [2,5]. As such, direct overt encephalopathy is an unusual presentation of lead poisoning [2,6-8]. Patients with BLL > 70 mcg/dL with evidence of encephalopathy should receive chelation with two agents- dimercaprol (British antilewisite, BAL- 300-500 mg/m^2^/day intramuscularly in q4h doses x 3-5 days) and CaNa2EDTA (1,000-1,500 mg/m^2^/day continuous intravenous infusion x 5 days) [9]. D-penicillamine, although effective in chelating lead, is a third-line chelating agent due to high frequency of side effects like leukopenia, thrombocytopenia, maculopapular rash, diarrhea, etc [9,10]. Due to resource-limited settings, we had to use D-penicillamine initially, followed by three-day course of CaNa2EDTA, nevertheless with a significant improvement. The patient returned to a near-normal state as in the pre-toxicity phase. Such reversal of encephalopathy has been reported in very few cases [7,8]. Perlstein et al. reported neurological sequelae of 59 patients presenting as lead encephalopathy, of whom only 18% (11) had normal neurological outcome and 82% (48) had neurological sequelae, 38% (23) having profound mental retardation with many remaining in a vegetative state, 54% (32) developed seizure disorder, 13% (8) developed cerebral palsy and 6% (4) developed optic atrophy despite chelation therapy [4].

## Conclusions

Lead poisoning remains a significant health problem even today, particularly in the industrial belts of the country. Early recognition and management are of paramount importance in its outcome. It is essential to evaluate the parents' occupation and the environmental exposure for a child with encephalopathy. A simple history taking, clinical examination, and X-ray of long bones can provide a clue to the diagnosis. Emergency care like seizure control and management of raised intracranial pressure can be life-saving in acute lead encephalopathy. Chelating agents like dimercaprol and CaNa2EDTA should be given as soon as possible. However, in non-availability in resource-limited settings, third-line agent D-penicillamine can be given with monitoring for its toxicity. Lead encephalopathy, by and large, leads to neurological sequelae, but its early recognition and management can aid in preventing it.
